# A preliminary probabilistic nomogram model for predicting renal arteriolar damage in IgA nephropathy from clinical parameters

**DOI:** 10.3389/fimmu.2024.1435838

**Published:** 2024-07-01

**Authors:** Huifang Wang, Xiaodan Zhang, Li Zhen, Hang Liu, Xuemei Liu

**Affiliations:** Department of Nephrology, The Affiliated Hospital of Qingdao University, Qingdao, China

**Keywords:** IgA nephropathy, arteriolar damage, kidney biopsy, predictive factors, nomogram model

## Abstract

**Background:**

IgA nephropathy (IgAN) is a significant contributor to chronic kidney disease (CKD). Renal arteriolar damage is associated with IgAN prognosis. However, simple tools for predicting arteriolar damage of IgAN remain limited. We aim to develop and validate a nomogram model for predicting renal arteriolar damage in IgAN patients.

**Methods:**

We retrospectively analyzed 547 cases of biopsy-proven IgAN patients. Least absolute shrinkage and selection operator (LASSO) regression and logistic regression were applied to screen for factors associated with renal arteriolar damage in patients with IgAN. A nomogram was developed to evaluate the renal arteriolar damage in patients with IgAN. The performance of the proposed nomogram was evaluated based on a calibration plot, ROC curve (AUC) and Harrell’s concordance index (C-index).

**Results:**

In this study, patients in the arteriolar damage group had higher levels of age, mean arterial pressure (MAP), serum creatinine, serum urea nitrogen, serum uric acid, triglycerides, proteinuria, tubular atrophy/interstitial fibrosis (T1–2) and decreased eGFR than those without arteriolar damage. Predictors contained in the prediction nomogram included age, MAP, eGFR and serum uric acid. Then, a nomogram model for predicting renal arteriolar damage was established combining the above indicators. Our model achieved well-fitted calibration curves and the C-indices of this model were 0.722 (95%CI 0.670–0.774) and 0.784 (95%CI 0.716–0.852) in the development and validation groups, respectively.

**Conclusion:**

With excellent predictive abilities, the nomogram may be a simple and reliable tool to predict the risk of renal arteriolar damage in patients with IgAN.

## Introduction

IgA nephropathy (IgAN) is the most common primary glomerulonephritis worldwide, and it is also a significant contributor to the development of end stage renal disease (ESRD). It has been reported that 25–40% of IgAN patients develop ESRD within 10 to 20 years (1, 2). Patients with IgAN exhibit a wide range of clinical symptoms, from asymptomatic hematuria and proteinuria to heavy proteinuria, and further to acute renal failure and chronic renal insufficiency (3). Disease progression and prognosis vary among individuals, given the clinical and pathological diversity of IgAN (4).

It has been realized that renal arteriolar damage is also common in IgAN. Although the original Oxford classification did not identify the predictive value of renal arteriolar damage for treatment and prognosis in patients with IgAN (5), a growing number of studies have found that renal arteriolar damage is an independent prognostic risk factor in patients with IgAN (6). Some studies suggest incorporating arteriolar damage into the classification to more comprehensively assess and predict the clinical progression and treatment response in IgAN (7). Currently, there is no doubt that renal biopsy remains the gold standard for diagnosis, and plays a pivotal role in the assessment and prognosis of renal damage in IgAN patients. Nevertheless, renal biopsy poses considerable risks of hemorrhage and infection, and it is an invasive procedure and its indications remain controversial. This indicates the necessity of a non-invasive approach to evaluate the severity of renal damage. Simple tools for predicting renal arteriolar damage in patients with IgAN are limited.

In recent years, statistical prediction models have been widely used in investigations of clinical diseases. In this study, we constructed a nomogram to predict the renal arteriolar damage in patients with IgAN using a series of blood and urine test results, which helped physicians make early clinical decisions while avoiding unnecessary renal biopsy.

## Materials and methods

### Study population and design

This retrospective cohort study was conducted at the Affiliated Hospital of Qingdao University between January 2015 and June 2023. Patients diagnosed with IgAN by renal biopsy were included in the study. Patients were eligible for inclusion if they met the following criteria: (1) had a diagnosis of primary IgAN through biopsy; (2) presence or absence of renal arteriolar damage. The exclusion criteria were as follows: (1) age at IgAN diagnosis < 18 years; (2) secondary IgAN, such as systemic lupus erythematosus, Henoch-Schonlein purpura nephritis, hepatitis B virus-related glomerulonephritis, or diabetic nephropathy; (3) no complete clinical data at baseline; and (4) less than 8 glomeruli or less than 3 vessels in renal biopsy specimens for light microscopic examination or missing renal pathology reports. Ultimately, 547 patients with IgAN were included and randomly divided into the development and validation cohorts at a ratio of 7:3. Due to the absence of personal identifiers in the database and the retrospective, observational nature of the study design, the requirement for informed consent was waived. The study protocol was in accordance with the provisions of the Declaration of Helsinki and approved by the Ethics Committee of the Affiliated Hospital of Qingdao University (IRB approval number: QYFY WZLL 28700).

### Clinical and laboratory data

The general information, clinical and laboratory examinations and histologic features of the patients included in this study were collected at the time of kidney biopsy. The clinical indicators included age, sex, BMI, blood pressure and mean arterial pressure (MAP). Laboratory data included hemoglobin, platelet counts, serum creatinine, serum uric acid, serum urea nitrogen, serum albumin, triglycerides, total cholesterol, low-density lipoprotein cholesterol (LDL-C), IgA, complement levels, proteinuria and urinary red blood cells (URBC). The estimated glomerular filtration rate (eGFR) was calculated using the Chronic Kidney Disease Epidemiology Collaboration equations (CKD-EPI) formula (8). The BMI was calculated as weight (kg)/height (m^2^). These data were used as key variables in the development of statistical nomogram prediction models.

### Renal pathology evaluation

Light microscopy and immunofluorescence were used to examine all IgAN specimens. Biopsies were scored according to the Oxford Classification Scoring System of IgAN (MEST-C score) (5, 9), including mesangial hypercellularity (M), endocapillary hypercellularity (E), segmental glomerulosclerosis (S), interstitial fibrosis/tubular atrophy (T), and cellular/fibrocellular crescents (C). The presence of arteriolar damage is defined as: a) the presence of arteriolar hyaline on the walls of any artery or arteriole, or b) intimal thickening that exceeds the thickness of the media in the same vascular segment. At least two pathologists independently evaluated the histopathological manifestations.

### Statistics

Normally distributed variables were presented as mean ± standard deviation (SD) and compared using Student’s t tests, and non-normally distributed variables were reported as median (interquartile range [IQR]) and compared using Mann–Whitney U tests. Categorical variables were presented as numbers and percentages using the Chi-square test or Fisher’s exact test. R version 4.0.3 (R Foundation for Statistical Computing) and SPSS version 25 were used to analyze the data.

In statistical tests, continuous variables with large coefficients of variation were log-transformed to reduce variability. The least absolute shrinkage and selection operator (LASSO) method and logistic regression were employed to select the most relevant predictive features associated with renal arteriolar damage in IgAN. Features with nonzero coefficients in the LASSO regression model were selected (10). Then, multivariable logistic regression analysis (stepwise regression according to the Akaike information criterion [AIC]) was used to build a predictive model by incorporating the features selected in the LASSO regression model. The features were considered as odds ratios (OR) with 95% confidence intervals (*CI*) and as *P*-value. The statistical significance levels were all two sided. Sociodemographic variables with a *P*-value of <0.05 were included in the model, whereas variables associated with disease characteristics were included (11). All potential predictors were applied to develop a predictive model for renal arteriolar damage risk in patients with IgAN by using the cohort (12).

Calibration curves were plotted to assess the calibration of the nonadherence nomogram (13). The discrimination was quantified by the area under the receiver operating characteristic (ROC) curve (AUC). Harrell’s C-index was measured to quantify the discrimination performance of the nonadherence nomogram, and calibration with 1000 bootstrap samples was performed to decrease the overfit bias. Both the models were validated in the testing cohort. A nomogram was constructed based on the regression equation of the preferable model. For all statistical analyses, *P*-values < 0.05 were considered statistically significant.

## Results

### Clinicopathological characteristics of the included patients

This study included 547 eligible patients with IgAN who were diagnosed with primary IgAN through biopsy. Among the patients, 263 (48.1%) were female and the median age was 40 (32, 52) years. All patients were from the Chinese Han population. The presence of arteriolar damage was 40.20%. There were no significant differences between the development and validation cohorts in baseline data (*p* > 0.05). The characteristics of the study population are summarized in **Table 1**.

**Table 1:** Baseline characteristics of the IgAN patients in the development and validation cohorts.

Variables	Total (*n*=547)	Development cohort (*n*=383)	Validation cohort (*n*=164)	*P* value
Age, years (median, IQR)	40.00(32.00, 52.00)	40.00(32.00, 51.00)	41.00(32.00, 53.75)	0.337
Female, n (%)	263.00(48.10)	184.00(48.00)	79.00(48.20)	0.978
Body mass index, kg/m^2^ (median, IQR)	24.70(22.70, 27.70)	24.70(22.60, 27.40)	24.80(22.80, 27.98)	0.281
Systolic BP, mmHg (median, IQR)	136.00(126.00, 147.00)	136.00(125.00, 148.00)	135.00(127.00, 146.00)	0.667
Diastolic BP, mmHg (median, IQR)	84.00(76.00, 92.00)	84.00(76.00, 92.00)	83.00(77.00, 91.75)	0.913
MAP, mmHg (median, IQR)	101.33(93.00,109.67)	101.33(93.33, 110.00)	100.83(93.50, 108.50)	0.830
Laboratory				
Hemoglobin, g/L (mean ± SD)	132.71 ± 20.08	132.13 ± 20.86	132.95 ± 19.75	0.663
Platelet counts, 10^9^/L (median, IQR)	240.00(204.00,285.00)	240.00(204.00, 285.00)	239.00(203.75, 281.75)	0.811
Serum uric acid, μmol/L (median, IQR)	364.50(313.00, 423.00)	366.00(317.00, 424.70)	360.15(309.25, 422.75)	0.853
Serum creatinine, μmol/L (median, IQR)	85.00(68.00, 112.30)	85.00(68.30, 111.90)	86.40(67.00, 114.53)	0.792
Serum urea nitrogen, mmol/L (median, IQR)	5.65(4.57, 7.53)	5.55(4.56, 7.49)	5.93(4.60, 7.84)	0.250
eGFR, ml/min/1.73m^2^ (median, IQR)	83.54(64.74, 105.70)	84.49(65.31, 106.97)	82.06 (60.04, 102.65)	0.242
Serum albumin, g/L (median, IQR)	37.23(33.52, 40.40)	37.35(33.60, 40.13)	37.11(33.40, 40.79)	0.977
Triglycerides, mmol/L (median, IQR)	1.41(0.99, 2.06)	1.45(0.97, 2.09)	1.37(1.02, 1.92)	0.690
Total cholesterol, mmol/L (median, IQR)	4.80(4.11, 5.73)	4.78(4.10, 5.67)	4.81(4.17, 5.78)	0.529
LDL-C, mmol/L (median, IQR)	2.97(2.40, 3.65)	2.90(2.38, 3.58)	3.03(2.40, 3.73)	0.272
IgA (g/L)	3.02(2.42, 3.65)	3.02(2.43, 3.70)	3.01(2.42, 3.66)	0.534
Complement 3 (g/L) (median, IQR)	1.05(0.91, 1.20)	1.04(0.91, 1.19)	1.08(0.91, 1.24)	0.088
Complement 4 (g/L) (median, IQR)	0.25(0.21, 0.30)	0.25(0.21, 0.30)	0.24(0.21, 0.29)	0.301
URBC≥30/uL, n (%)	273.00(50.10)	184.00(48.00)	89.00(54.30)	0.182
Proteinuria (g/day) (median, IQR)	1.30(0.65, 2.52)	1.27(0.68, 2.43)	1.40(0.61, 2.69)	0.614
Histological characteristics				
M1 (%)	404.00(73.90)	284.00(74.20)	120.00(73.20)	0.811
E1 (%)	109.00(19.90)	74.00(19.30)	35.00(21.30)	0.588
S1 (%)	290.00(53.00)	203.00(53.00)	87.00(53.00)	0.992
T1–2 (%)	78.00(14.30)	56.00(14.60)	22.00(13.40)	0.712
C1–2 (%)	66.00(12.10)	48.00(12.50)	18.00(11.00)	0.608
Arteriolar damage, n (%)	220.00(40.20)	144.00(37.60)	76.00(46.30)	0.076

Data are presented as the mean ± standard deviation (SD), median (interquartile range), or number (percentage).

BP, blood pressure; eGFR, estimated glomerular filtration rate; LDL-C, Low-density lipoprotein cholesterol; MAP, mean arterial pressure; URBC, urinary red blood cell.

### Characteristics in IgAN patients with arteriolar damage

Of the 383 patients in the development cohort, 144 patients (37.6%) were in the arteriolar damage group and 239 patients (62.4%) were in the non-arteriolar damage group. The median age of the recipients was 40.00 (32.00, 51.00) years and 48% of the patients were females in the development cohort. **Table 2** presents the baseline clinical characteristics of the two groups. Patients in the arteriolar damage group had higher levels of age, MAP, serum creatinine, serum urea nitrogen, serum uric acid, triglycerides and proteinuria than those without arteriolar damage (*p*< 0.05). The eGFR was significantly lower in IgAN patients with arteriolar damage than those without arteriolar damage (*P* < 0.05). The tubular atrophy/interstitial fibrosis (T1–2) was more severe in patients with arteriolar damage compared to those without arteriolar damage (*P* < 0.05). However, there were no differences in other factors in **Table 1** between IgAN patients with and without arteriolar damage (*P* > 0.05).

**Table 2:** Clinical characteristics of IgAN patients with or without arteriolar damage in development cohort.

Variables	Total (*n* =383)	Any arteriolar damage (*n* =144)	Non-arteriolar damage (*n* =239)	*P* value
Age, years (median, IQR)	40.00(32.00, 51.00)	45.00(36.25, 55.75)	37.00(31.00, 47.00)	<0.001
Female, n (%)	184.00(48.00)	62.00(43.10)	122.00(51.00)	0.130
Body mass index, kg/m^2^ (median, IQR)	24.70(22.60, 27.40)	25.45(23.23, 28.03)	24.00(22.00, 26.80)	0.001
Systolic BP, mmHg (median, IQR)	136.00(125.00, 148.00)	141.00(130.00, 152.50)	133.00(122.00, 144.00)	<0.001
Diastolic BP, mmHg (median, IQR)	84.00(76.00, 92.00)	87.00(80.00, 94.00)	82.00(73.00, 91.00)	<0.001
MAP, mmHg (median, IQR)	101.33(93.33, 110.00)	104.67(98.00, 112.33)	97.33(91.00, 108.33)	<0.001
Laboratory				
Hemoglobin, g/L (mean ± SD)	132.13 ± 20.86	133.57 ± 21.10	132.58 ± 18.93	0.635
Platelet counts, 10^9^/L ((median, IQR)	240.00(204.00, 285.00)	236.00(204.00, 282.00)	242.00(204.00, 288.00)	0.391
Serum uric acid, μmol/L ((median, IQR)	366.00(317.00, 424.70)	380.50(327.92, 449.50)	358.00(305.00, 409.00)	<0.001
Serum creatinine, μmol/L (median, IQR)	85.00(68.30, 111.90)	91.00(72.10, 123.73)	83.20(67.00, 110.29)	0.008
Serum urea nitrogen, mmol/L (median, IQR)	5.55(4.56, 7.49)	6.02(4.83, 8.10)	5.29(4.41, 7.18)	0.004
eGFR, ml/min/1.73m^2^ (median, IQR)	84.49(65.31, 106.97)	75.04(54.76, 96.05)	90.32(71.22, 110.29)	<0.001
Serum albumin, g/L (median, IQR)	37.35(33.60, 40.13)	36.82(32.66, 40.22)	37.60(34.39, 40.13)	0.423
Triglycerides, mmol/L (median, IQR)	1.45(0.97, 2.09)	1.55(1.09, 2.13)	1.37(0.91, 2.08)	0.038
Total cholesterol, mmol/L (median, IQR)	4.78(4.10, 5.67)	4.76(4.10, 5.81)	4.80(4.07, 5.66)	0.954
LDL-C, mmol/L (median, IQR)	2.90(2.38, 3.58)	2.84(2.40, 3.56)	2.92(2.38, 3.65)	0.791
IgA (g/L)	3.02(2.43, 3.70)	3.07(2.41, 3.87)	3.00(2.47, 3.60)	0.438
Complement 3 (g/L) (median, IQR)	1.04(0.91, 1.19)	1.05(0.93, 1.18)	1.04(0.91, 1.20)	0.856
Complement 4 (g/L) (median, IQR)	0.25(0.21, 0.30)	0.25(0.22, 0.30)	0.25(0.21, 0.31)	0.767
URBC≥30/uL, n (%)	184.00(48.00)	60.00(41.70)	124.00(51.90)	0.053
Proteinuria (g/day) (median, IQR)	1.27(0.68, 2.43)	1.49(0.79, 2.58)	1.11(0.61, 2.29)	0.039
Histological characteristics				
M1 (%)	284.00(74.20)	111.00(77.10)	173.00(72.40)	0.309
E1 (%)	74.00(19.30)	33.00(22.90)	41.00(17.20)	0.167
S1 (%)	203.00(53.00)	77.00(53.50)	126.00(52.70)	0.886
T1–2 (%)	56.00(14.60)	28.00(19.40)	28.00(11.70)	0.038
C1–2 (%)	48.00(12.50)	14.00(9.70)	34.00(14.20)	0.197

Data are presented as the mean ± standard deviation (SD), median (interquartile range), or number (percentage).

BP, blood pressure; eGFR, estimated glomerular filtration rate; LDL-C, Low-density lipoprotein cholesterol; MAP, mean arterial pressure; URBC, urinary red blood cell.

### Characteristics selection

**Figure 1:**
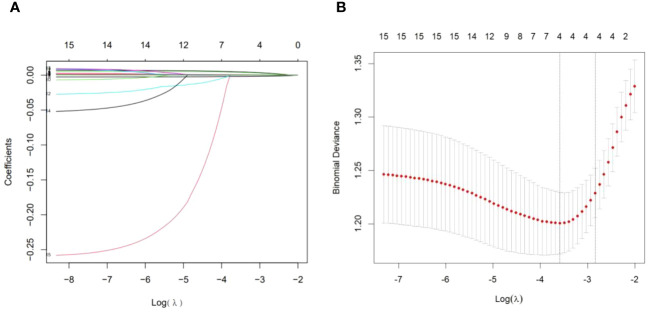
Demographic and clinical feature selection using the LASSO binary logistic regression model. **(A)** Optimal parameter (lambda) selection in the LASSO model used fivefold cross-validation via minimum criteria (10, 14). The partial likelihood deviance (binomial deviance) curve was plotted versus log (lambda). Dotted vertical lines were drawn at the optimal values using the minimum criteria and the 1 SE of minimum criteria (1-SE criteria). **(B)** LASSO coefficient profiles of 17 features. A coefficient profile plot was produced against the log (lambda) sequence. A vertical line was drawn at the value selected using fivefold cross-validation, where the optimal lambda resulted in four features with nonzero coefficients. LASSO, least absolute shrinkage and selection operator; SE, standard error.

In terms of clinical features, we used LASSO regression to identify the main variables related to arteriolar damage in IgAN patients, and a total of 4 variables were screened out of 17 features (**Figures 1A, B**). The variables included age, MAP, eGFR and serum uric acid (**Table 3**).

**Table 3:** Prediction factors for arteriolar damage in IgAN patients.

Intercept and variable	Prediction model		
	*β*	*OR* (95% *CI*)	*P* value
Age, each year increase	0.030	1.030 (1.012–1.050)	0.002
MAP, each mmHg increase	0.027	1.028 (1.010–1.046)	0.003
eGFR, each ml/min/1.73m^2^ increase	-0.011	0.989 (0.979–0.998)	0.020
Serum uric acid, each μmol/L increase	0.004	1.004 (1.001–1.008)	0.007

β is the regression coefficient.

eGFR, estimated glomerular filtration rate; MAP, mean arterial pressure.

### Development of an individualized prediction model

**Figure 2:**
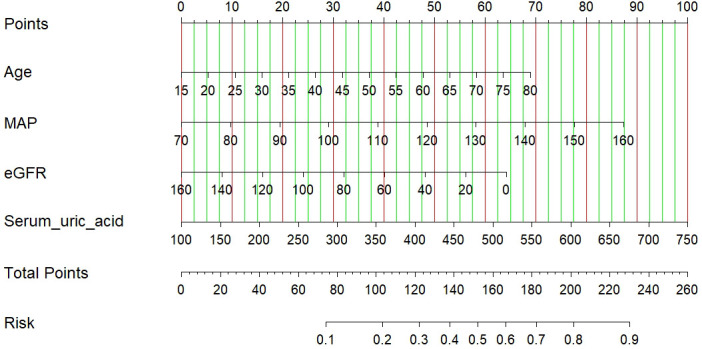
A nomogram of the LASSO model. The nomogram was developed with age, MAP, eGFR and serum uric acid. To use the nomogram, draw a line perpendicular from the corresponding axis of each risk factor until it reaches the top line labeled “Points”. Sum up the number of points for all risk factors then draw a line descending from the axis labeled “Total points” until it determined the probabilities of arteriolar damage. eGFR, estimated glomerular filtration rate.

The results of the logistic regression analysis among the age, MAP, eGFR and serum uric acid are shown in **Table 3**. A model that incorporated the above independent predictors was developed and presented as a nomogram (**Figure 2**). The scores of different variables were obtained on the vertical line on the nomogram, after which the total risk score was calculated by adding the scores of all variables.

### Validation of prediction model

**Figure 3:**
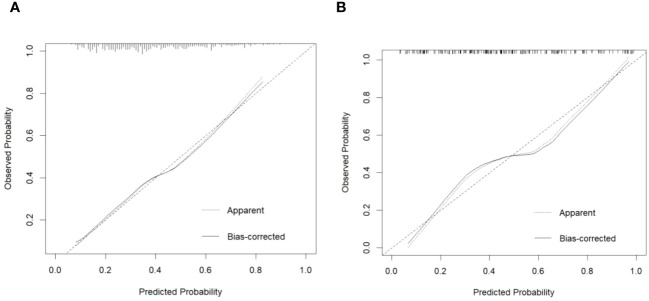
Calibration plot of predicted probability of arteriolar damage predicted by the nomogram model vs. observed probability in the development cohort **(A)** and validation cohort **(B)**. The diagonal dotted line represents a perfect prediction by an ideal model. The solid line represents the performance of the nomogram, of which a closer fit to the diagonal dotted line represents a better prediction.

**Figure 4:**
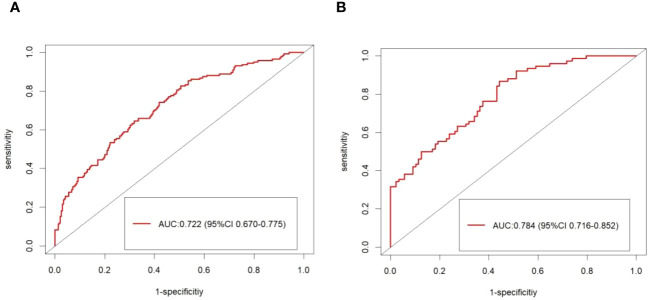
The ROC curve of the model forecasting the presence of arteriolar damage. The area under the ROC curve (AUC) were 0.722 (95% CI: 0.670–0.775) in the development cohort **(A)** and 0.784 (95% CI: 0.716–0.852) in the validation cohort **(B)**.

The calibration plot of the model demonstrated good consistency between the development and the validation groups for the prediction of arteriolar damage (**Figure 3**). According to ROC curve (AUC), the AUC value was 0.722 (95% CI: 0.670–0.775) in the development cohort and 0.784 (95% CI: 0.716–0.852) in the validation cohort (**Figure 4**), indicating that the model had medium discrimination. The C-index of the prediction model was 0.722 (95%CI 0.670–0.774). We further performed internal validation on the nomogram by bootstrapping validation, and the C-index was 0.784 (95%CI 0.716–0.852), which indicated that the model had a relatively great predictive discrimination.

## Discussion

Arteriolar damage is a common lesion identified by renal biopsy in patients with IgAN, and it has more clinical and pathological risk factors that may lead to a poor prognosis for IgAN (6). Therefore, there is an urgent need for a predictive model to help clinicians accurately distinguish arteriolar damage in patients with IgAN. In this study, we used a large cohort of 547 individuals to establish a tool to predict the risk of renal arteriolar damage in patients with IgAN to guide clinical management. The nomogram serves as a visually straightforward predictive tool, utilized for forecasting specific clinical outcomes (15). The C-index from the internal validation indicated that the model possessed good discriminatory and calibration capabilities. Furthermore, we conducted an analysis of the ROC curves to validate the accuracy of this nomogram. The nomogram suggests that age, MAP, eGFR and serum uric acid may be used as independent risk factors for renal arteriolar damage in patients with IgAN.

Until now, there have been limited comprehensive investigations into the detailed clinicopathological features and risk factors associated with vascular lesions in patients with IgAN. In our study, we found that 40.20% of patients had arteriolar damage, suggesting that arteriolar damage in IgAN were very common. The arteriolar damage group had higher age, MAP, serum creatinine, serum uric acid, triglycerides, proteinuria and decreased eGFR than those without arteriolar damage (*P* < 0.05). In addition, in our study, arteriolar damage was also associated with higher scores of tubular atrophy/interstitial fibrosis (T1–2) in patients with IgAN, indicating that arteriolar damage reflects chronic lesions. Similar results had been reported in some previous studies (6, 16, 17). These correlations may suggest a common pathogenesis in arteriolar damage and renal interstitial histopathological lesions. The initial mechanism of vascular damage may involve glomerular inflammatory changes in patients with IgAN. During the process of glomerular injury, inflammatory cells and mediators cause a phenotypic transformation of interstitial and tubular epithelial cells into myofibroblasts, leading to interstitial fibrosis, tubular atrophy, and vascular lesions. Concurrently, vascular lesions affect the blood supply to the glomeruli and contribute to the vicious cycle of damage that affects these tissues. Furthermore, tubular atrophy/interstitial fibrosis had been demonstrated to be an independent risk factor for renal outcomes (18). The correlation between arteriolar damage and tubular atrophy/interstitial fibrosis may also indicate poor renal prognosis in patients with arteriolar damage.

We found that arteriolar damage was strongly associated with higher MAP. It is well known that hypertension can cause atherosclerosis and arteriolar hyaline in primary renal parenchymal disease, contributing to progressive renal insufficiency. Arteriolar damage may be caused by hemodynamic changes induced by hypertension. Higher blood pressure had been reported as a major risk factor for renal outcome (17, 19), which indirectly confirmed that vascular lesions are also a key factor affecting the prognosis of IgAN. Our study also showed that some patients had arteriolar damage without presenting with hypertension clinically, suggesting that vascular changes may precede the onset of hypertension. This further highlights the importance of evaluating arteriolar damage, as it may predict the development of hypertension and improve prognosis.

Hyperlipidemia is another significant factor contributing to arteriolar damage, in addition to the hemodynamic alterations induced by hypertension. Our study also found that compared to patients without arteriolar damage, patients with arteriolar damage had higher levels of serum triglycerides. Therefore, hyperlipidemia is potentially a contributing factor to arteriolar injury in patients with IgAN, with prevailing views suggesting that such damage is linked to a maladaptive inflammatory response (20). A multitude of inflammatory cytokines have been implicated in the onset and advancement of IgAN (21). Further investigation is needed to identify the specific causes of arteriolar damage in patients with IgAN.

Our data also showed that arteriolar damage correlates with decreased eGFR in patients with IgAN, and that renal insufficiency is independently associated with arterial lesions. The study by Bos et al. (22) showed that a decline in renal function is accompanied by intimal proliferation of renal arterioles, even in the absence of hypertension. Moreover, lower eGFR is associated with poor outcome in IgAN (19, 23). In a retrospective study, Zhang et al. demonstrated that the presence of vascular lesions was associated with poorer renal outcomes (17). Similarly, Russo et al. reported that patients with arterial disease were at a higher risk of death or progression to ESRD (24). However, the original Oxford study showed that arterial lesions did not correlate with the rate of decline in renal function. Additionally, our study found that increased serum uric acid is also an independent risk factor for renal arteriolar damage in patients with IgAN. However, the increase in serum uric acid levels may be attributed to a low eGFR leading to reduced uric acid excretion. Previous studies have demonstrated the association between serum uric acid and renal arteriolar damage in patients with chronic kidney disease (24, 25). Elevated serum uric acid can lead to oxidative stress and endothelial dysfunction, which in turn can cause renal vasoconstriction, glomerular hypertension, and a reduction in renal blood flow. This can activate the renin-angiotensin system (RAS) and induce pre-glomerular arteriolar disease characterized by arteriolar wall thickening and hyalinosis, thereby promoting ischemia (26). Therefore, clinical research is still needed to explore the relationship between arteriolar damage and prognosis in patients with IgAN.

Our study had some limitations. First, our study was a retrospective study conducted at a single center, not as effective as a prospective study, and did not include long-term follow-up observation. Second, our data were derived from patients who underwent renal biopsies, and some patients were initially excluded for not having a renal biopsy for certain reasons. Third, the nomogram has not been externally validated in IgAN populations from different regions and countries, and it may require additional validation through a prospective multicenter study involving diverse cohorts.

## Conclusion

Based on the three risk factors of age, MAP, eGFR and serum uric acid, a nomogram model was constructed to predict the risk of renal arteriolar damage in patients with IgAN based on laboratory tests. The model exhibited high accuracy, discrimination and predictive ability, suggesting its promising applicability in the clinical identification and medical management of patients at high risk.

## Data availability statement

The raw data supporting the conclusions of this article will be made available by the authors, without undue reservation.

## Ethics statement

The studies involving humans were approved by The Ethics Committee of the Affiliated Hospital of Qingdao University (IRB approval number: QYFY WZLL 28700). The studies were conducted in accordance with the local legislation and institutional requirements. Due to the absence of personal identifiers in the database and the retrospective, observational nature of the study design, the requirement for informed consent was waived.

## Author contributions

HW: Writing – original draft, Writing – review & editing. XZ: Data curation, Methodology, Writing – review & editing. LZ: Investigation, Validation, Writing – review & editing. HL: Data curation, Validation, Writing – review & editing. XL: Supervision, Writing – review & editing.
